# New Roles of Glycosaminoglycans in α-Synuclein Aggregation in a Cellular Model of Parkinson Disease

**DOI:** 10.1371/journal.pone.0116641

**Published:** 2015-01-24

**Authors:** Sonia Lehri-Boufala, Mohand-Ouidir Ouidja, Véronique Barbier-Chassefière, Emilie Hénault, Rita Raisman-Vozari, Laure Garrigue-Antar, Dulce Papy-Garcia, Christophe Morin

**Affiliations:** 1 Université Paris-Est Créteil, Laboratoire CRRET-EAC CNRS 7149, 61 Avenue de Général de Gaulle, 94010, Créteil, France; 2 CNRS UMR 7225, Hôpital de la Salpêtrière-Bâtiment, ICM (Centre de Recherche de l’Institut du Cerveau et de la Moelle épinière), CRICM, Thérapeutique Expérimentale de la Neurodégénérescence, Université Pierre et Marie Curie, UPMC, 75651, Paris, France; University of Leipzig, GERMANY

## Abstract

The causes of Parkinson disease (PD) remain mysterious, although some evidence supports mitochondrial dysfunctions and α-synuclein accumulation in Lewy bodies as major events. The abnormal accumulation of α-synuclein has been associated with a deficiency in the ubiquitin-proteasome system and the autophagy-lysosomal pathway. Cathepsin D (cathD), the major lysosomal protease responsible of α-synuclein degradation was described to be up-regulated in PD model. As glycosaminoglycans (GAGs) regulate cathD activity, and have been recently suggested to participate in PD physiopathology, we investigated their role in α-synuclein accumulation by their intracellular regulation of cathD activity. In a classical neuroblastoma cell model of PD induced by MPP^+^, the genetic expression of GAGs-biosynthetic enzymes was modified, leading to an increase of GAGs amounts whereas intracellular level of α-synuclein increased. The absence of sulfated GAGs increased intracellular cathD activity and limited α-synuclein accumulation. GAGs effects on cathD further suggested that specific sequences or sulfation patterns could be responsible for this regulation. The present study identifies, for the first time, GAGs as new regulators of the lysosome degradation pathway, regulating cathD activity and affecting two main biological processes, α-synuclein aggregation and apoptosis. Finally, this opens new insights into intracellular GAGs functions and new fields of investigation for glycobiological approaches in PD and neurobiology.

## INTRODUCTION

Parkinson’s disease (PD) is one of the most common neurodegenerative diseases affecting almost 1% of the population worldwide and is mainly characterized by the death of dopaminergic neurons in the *substantia nigra*. Even though the cause of PD remains mysterious, several lines of evidence support mitochondrial dysfunctions [1] and α-synuclein accumulation in Lewy bodies (LB) as major disease events. Although the formation of LB and their role in dopaminergic neuron death remain elusive [2, 3], the biological function of α-synuclein is not fully understood. However, this protein is abundantly expressed in pre-synaptic terminals and seems to be involved in the regulation and the maintenance of dopaminergic cells homeostasis. Importantly, α-synuclein over-expression has been associated to several traits of PD in animal models with mitochondrial complex I dysfunctions, whereas α-synuclein null mice display striking resistance to the degeneration of dopaminergic neurons and dopamine release induced by the neurotoxin MPTP [4, 5]. The central adverse feature of α-synuclein over-expression is its tendency to misfold and to subsequently aggregate in oligomers suspected to induce toxicity in neurons, as described with other proteins aggregates [6]. Once accumulated, α-synuclein spontaneously aggregates and promotes inclusion formation which is suggested to lead to LB. The accumulation of α-synuclein and its subsequent aggregation have been associated with a deficiency in protein processing considered as one of the potential starting point of the disease [7]. While this remains to be confirmed and the precise mechanism of α-synuclein degradation is not yet fully understood, several studies have suggested that all major cellular degradation pathways are involved in its catabolism [8, 9]. In PD, dysfunctions of the ubiquitin-proteasome system (UPS) could lead to the accumulation of the ubiquitinylated α-synuclein with its concomitant aggregation into inclusion bodies, which would finally induce neuronal death [10]. Beside this, the autophagy-lysosomal pathway (ALP) is progressively diminished with age and the impairment of its protein processing capacity is associated with a higher risk of neurodegeneration [11, 12, 13]. Interestingly, recent studies suggest that under normal conditions, the UPS is the main degradation pathway for α-synuclein, while the ALP is recruited when the α-synuclein expression increases, such as in pathologic conditions [11]. Among the ALP pathway protagonists, the lysosomal aspartic protease cathepsin D (cathD) was described as the main α-synuclein degrading enzyme [14], able to prevent α-synuclein aggregation and toxicity [15, 16]. Thus, in a rhesus monkey model of PD, over-expression of cathD and the increased number of lysosomes were correlated with apoptotic processes [17]. However, these findings highlighted the discrepancy between the presence of cathD and its inefficiency to prevent α-synuclein accumulation, suggesting the presence of unidentified endogenous inhibitor(s). It has long been known that cathD activity is inhibited by endogenous sulfated polysaccharides, i.e. glycosaminoglycans (GAGs), which are present in lysosomes where they are processed after endocytosis [18, 19]. In previous studies, we showed that GAGs and their synthetic mimetics can effectively modulate cathD activity, protect mitochondria, and inhibit apoptosis-induced oxidative stress [19, 20]. This suggests the potential role of GAGs in pathologies where such processes occur. Interestingly, a typical mucopolysaccharidosis mutation in α-*N*-acetylglucosaminidase (NAGLU) gene, leading to lysosomal impairment, GAGs accumulation, with concomitant α-synuclein aggregation, was recently described in PD patients [21]. Heparan sulfates (HS), the most structurally diverse sub-family of GAGs processed by NAGLU, have been involved in the aggregation processes of proteins including Aβ peptide, Tau, PrP and α-synuclein [6]. Furthermore, we recently demonstrated that GAGs involvement is not limited to the classical aggregation process, but are needed in the cell uptake and propagation for tauopathy and synucleinopathy [22]. Tau and α-synuclein enter cells via macropinocytosis in which GAGs serve as primary receptor [23]. Then macropinosomes undergo traditional maturation and fuse with the lysosome [24].

Based on these data, we hypothesized that GAGs can modulate cathD activity which regulates α-synuclein degradation/accumulation in cells. In this report, we studied particular fates and functions of GAGs in a cellular model of PD implicating 1-methyl-4-phenylpyridinium ion (MPP^+^) stress to investigate whether GAGs can interact with cathD. We demonstrate for the first time that the biosynthesis and cellular contents of GAGs, including HS and chondroïtin sulfate (CS), are modified by MPP^+^ stress and that changes are associated with differential expression of some specific GAGs biosynthetic enzymes. We show that the cellular GAGs regulate cathD activity, and importantly, that their presence is required for α-synuclein intracellular accumulation. Altogether, these results suggest important regulatory roles for GAGs in protein degradation/accumulation, which may thus involve them in the physiopathology of neurodegenerative diseases.

## EXPERIMENTAL

### Chemicals and reagents

Dulbecco’s Minimal Essential medium (DME), foetal bovine serum (FBS), penicillin/streptomycin, trypsin-EDTA solution and phosphate buffered saline (PBS) were from GibcoBRL (France). 1-Methyl-4-phenylpyridinium (MPP+), 3-(4,5-Dimethylthiazol-2-yl)-2,5-diphenyltetrazolium bromide (MTT), chondroitin sulfate (CS) heparan sulfate (HS), heparin (Hep) and anti-CS antibody were from Sigma (France). Anti Bax and cathD antibodies were from Santa Cruz (USA). Caspase substrates Ac-DEVD-AFC, Ac-IETD-AFC and Ac-LEHD-AFC were purchased from Calbiochem (France). CathD substrate MCA-Gly-Lys-Pro-lle-Leu-Phe-Phe-Arg-Leu-Lys(DNP)-D-Arg-NH_2_ was purchased from Tebu-bio (France). Anti α-synuclein antibody was from Millipore (France) and anti HS 10E4 from Seikagaku (Japan). Fluorescent based enzymatic assays were performed in a micro-plate reader (TECAN infinite M1000).

### *In vitro* α-synuclein oligomerisation

*In vitro*, the spontaneous aggregation of α-synuclein (25 μg/mL) was evaluated by incubating the protein at 37°C during 24 h in the presence or not of commercial GAGs. Then the amounts of monomeric, oligomeric and aggregated forms of α-synuclein were estimated by western blot [5]. Briefly, samples were resolved on a 10% SDS-polyacrylamide gel and blotted onto PVDF blotting membranes. Membranes were washed with Tris-glycin buffer (25 mM Tris-HCl, 192mM glycin, pH 8) containing 0.2% Tween 20 and 5% bovine serum albumin to block the nonspecific protein binding. Membranes were incubated with monoclonal antibodies raised against human α-synuclein antibody in Tris-buffered saline containing 0.2% Tween 20 and 0.5% nonfat dry milk for 2h at room temperature), washed five times, and then incubated with horseradish peroxidase-conjugated rabbit anti-mouse IgG (1:5000) for 1.5 h at room temperature. α-synuclein was detected using the ECL method (Amersham Biosciences). Immunoblots were quantified by ImageJ software.

### Cell culture and treatment

Human neuroblastoma cells SH-SY5Y (ATCC CRL2266) were cultured in DMEM containing 4.5 g/L glucose, 10% heat FBS and 100 IU/mL penicillin/streptomycin, at 37°C in humidified 95% air with 7% CO_2_. For stress experiments, neuroblastoma cells were plated at a density of 2.5x10^4^ cells/mL. After 24 h incubation, stress was induced in the cells by treatment at 37°C for 6 h with 0.5 mM MPP^+^. Then, the medium was discarded and replaced by fresh medium supplemented or not with commercial GAGs (CS, HS or Hep). 24 h later, cells were subjected to morphological and biochemical evaluation. The synthesis of sulfated GAGs in cultured cells was inhibited with the metabolic inhibitor of sulfation, sodium chlorate (75 mM, 24 h) [22]. Medium was then discarded to induce the stress with MPP^+^ as previously described [25]. Endogenous cathD activity was inhibited by its specific inhibitor, pepstatin A (peps, 100 μM) by its addition in the medium during 24 h.

### Protein extraction

Cytosolic and mitochondrial protein extracts were performed for assessing cathD and Bax levels after washing cells with PBS. Extracts were prepared by an incubation of 5 min at 4°C in a buffer containing 250 mM sucrose, 70 mM KCl, 137 mM NaCl, 4.3 mM NaH_2_PO_4_, 1.4 mM KH_2_PO_4_, pH 7.2 and 100 mg/mL of digitonin. After centrifugation (10 min at 12 000 g), the supernatant corresponded to the cytosolic extract and the pellet to mitochondria fraction [19]. All western blot experiments were normalized with number of cells counted by MACSQuant flow cytometer Miltenyi as described in previous studies [19]. Succinate dehydrogenase-A (SDHA) and GAPDH were used as loading controls for mitochondrial and cytosolic protein extracts in western blots.

### Cathepsin D activity

Cathepsin D activity was assessed by measuring the hydrolysis of the fluorescent substrate MCA-Gly-Lys-Pro-lle-Leu-Phe-Phe-Arg-Leu-Lys(DNP)-D-Arg-NH_2_ (20 mM) at λex 328 nm and λem 393 nm, as previously described [19]. Endogenous cathD activity was measured in cell extracts subjected or not to MPP^+^ stress during 6 h followed by a treatment or not with commercial GAGs. CathD activity was normalized by cell number in each sample and expressed as percentage of the activity in normal cells. For chlorate experiments, total cell lysate fractions were used. In apoptosis experiments, cytosolic proteins were extracted 24 h after stress and used for the enzymatic cathD assay as previously described [19]. Briefly, the activity of cathepsin D was measured in cytosolic extracts obtained by incubating cells 5 min at 4°C in a buffer (250 mM sucrose, 70 mM KCl, 137 mM NaCl, 4.3 mM NaH_2_PO_4_,1.4 mM KH_2_PO_4_, pH 7.2) containing 100mg/ml of digitonin [26]. After a centrifugation of 10 min at 12 000g, supernatant and the pellet corresponding to the cytosolic extract and the mitochondrial fraction were stored at -20°C until use. GAGs extracted from stressed by MPP+ or normal neuroblastoma cells were incubated with commercial cathD (0.5 mM) during 1 h at 37°C in presence of specific substrate and then the fluorescence was measured as previously described. In all experiments, pepstatin A (peps, 0.3 mM), a specific cathD inhibitor, was used to estimate specific contributions of cathD to the monitored reaction.

The capacity of catD (50 mU/mL) to process α-synuclein was measured by incubating α-synuclein (25 μg/mL) at 37°C during 30 min, in the presence or not of different HS, CS and Hep quantities. The amount of the remaining non-degradated α-synuclein was estimated by western blot analysis and the effects of the different GAGs on the enzymatic degradation were compared.

### Assessment of respiratory chain function

Oxidative phosphorylation of intact cells and oxidation of mitochondrial substrate were estimated by polarography in a DW1 Clark oxygen electrode (Hansatech Instruments; Norfolk, UK) using 0.006% digitonin-permeabilized cells as previously described [26]. In this system, ADP addition causes a sudden burst of oxygen uptake when the ADP is converted into ATP, characterized by an actively respiring state (state 3) followed by a slower rate when total ADP has been phosphorylated to form ATP (state 4). The ratio [state 3 rate] / [state 4 rate], called the respiratory control index (RCI), indicates the tightness of the oxidative phosphorylation, referring to the respiratory chain functionality.

### Immunocytology

SH-SY5Y cells were stressed by MPP^+^ or not during 6 h and fixed in acetic acid 3% for 10 min at room temperature (RT). After three washes in PBS, cells were permeabilized with 0.02% Triton X-100 in 1X PBS, 10% FBS. Then, cells were immunostained for 1 h 30 at RT with a primary antibody against α-synuclein (1: 500), 10E4 antibody against HS (1: 200), antibody against CS (1: 200) or antibody against cathD (1: 200). After 3 washing in PBS, immunostaining was revealed by 1 h incubation with an anti-rabbit Fluo 546-conjugated secondary antibody (Molecular Probes, 1:200), an anti-goat Fluo 546-conjugated secondary antibody (Interchim, 1: 200) or anti-goat Fluo 488 or an anti-mouse Fluo 488-conjugated secondary antibody (Molecular Probes, 1: 200) depending on the first antibody and the second antibody for double staining. Cells were observed under confocal microscope (Zeiss Axio Observer Z.1). Negative control omitting the first antibody but in the presence of the second one did not show any signal for all the experiments.

### Extraction and characterization of GAGs

Cellular GAGs were extracted, isolated and characterized from SH-SY5Y cells subjected or not to MPP^+^ stress. At specified times, cells were treated with proteinase K (100 μg/mL) during 24 h at 56°C. The enzyme was then inactivated by increasing the temperature to 90°C during 30 min. DNA was eliminated by DNase digestion (7.5 U, 24 h at 37°C). Samples were diluted 1:1 with NaCl 4 M to abolish remaining GAG-peptides interactions. Then, samples were treated with cold trichloroacetic acid to 10% final concentration and lipids were removed by chloroform extraction (1:1). Samples were then dialyzed in buffer (50 mM Tris, 50 mM CH_3_COONa, 2 mM CaCl_2_, pH 8) twice during 3 h and then in water. After extraction, the sulfated polysaccharides were quantified by using the cationic dye 1, 9-dimethylmethylene blue (DMMB) as previously described [27]. Then, the amount of total GAGs per 10^6^ cells and the ratio CS/HS were measured using specific enzymatic or chemical degradation as already described [28]. Briefly, HS was quantified by DMMB assay after digesting CS with chondroitinase ABC (20 mU, 37°C, 1 h 30). For CS quantification, a nitrous acid treatment was used to selectively depolymerized HS from total GAGs samples. This treatment consisted in adding 0.25 M NaNO_2_ (100 μL) and acetic acid 33% (100 μL) to the samples, in order to carry out chemical digestion for 1 h at RT, followed by the addition of ammonium sulfamate (100 μL, 14%) to stop the reaction. After HS digestion, the DMMB assay was performed, then remaining CS were quantified and expressed as percentage of total GAG amount per 10^6^ cells.

For HS or CS disaccharides analysis, after the selected digestion, the degraded GAGs were eliminated by dialysis in buffer (50 mM Tris, 50 mM CH_3_COONa, 2 mM CaCl_2_, pH 8) twice during 6 h and then in water. After lyophilization, specific disaccharides were obtained by sample treatment with either heparinase I, II, and III (AMSBio, U.K.) cocktail (0.25 mU each, 24 h, 37°C) for HS disaccharides analysis or chondroitinase ABC (Sigma-Aldrich) (20 mU, 90 min, 37°C) for CS disaccharides analysis. Samples were analyzed by HPLC as previously described [29] with some modifications. Briefly, 50 μL of sample was loaded onto a Propac PA-1 (Dionex) strong-anion exchange column eluted by a NaCl-solvent gradient (0.1 to 1 M). Post-column in-line modification was realized by mixing 2-cyanoacetamide solution (2% v/v) and 250 mmol/L NaOH supplied both at 0.25 mL/min. The mixture passed through a reaction coil set in an oven at 120°C, followed by a cooling coil and then fluorescence was monitored (λ = 346 nm excitation, λ = 410 nm emission). Areas under curve were measured and the percentage of each disaccharide in sample was calculated relative to external standards.

### Real time RT-PCR

Total RNA were extracted from the neuroblastoma cells which were stressed or not by MPP^+^(6 h). The extraction was performed with the Qiagen “RNeasy Mini Kit” and quantified with a spectrophotometer UV (Jasco, France), where 1 absorbance unit corresponds to a concentration of 40 mg/mL of total RNA. Then, the reverse transcription reaction was performed on 1 μg of total RNA by using oligo dT primers and the Superscript III preamplification system (Invitrogen, France) according to the manufacturer’s instructions. Gene expression was quantified by real-time PCR using the LightCycler Fast DNA Master (Roche, France) with 2 μL cDNA corresponding to 200 ng total RNA, 4 mM magnesium chloride and 0.5 μM primer (final concentration, S1 Table). Briefly, quantitative PCR was performed for 45 cycles at 95°C for 15 sec, at the specific annealing temperature for 25 sec and at 72°C for 30 sec. Amplification specificity was checked using a melting curve following the manufacturer’s instructions. Results were analyzed with the LightCycler software v3.5 (Roche, France) using the second derivative maximum method to set the threshold cycle (Ct). As we previously described [28], the quantitative analysis was carried out using standard curves and were normalized using GeNorm software and methodology [30]. Three different housekeeping genes were used (Glutamine synthetase, GAPDH and α-tubulin).

### Data analysis

Group data are expressed as mean ± S.E.M. Comparisons among groups were made by ANOVA (F-test), Bonferroni adjusted t-tests were used for multiple group comparisons, and unpaired t-test was used for single comparisons. A two-tailed p<0.05 was selected to indicate a statistical significant difference. All values were calculated using GraphPad Prism 5.00 (GraphPad Software, San Diego, CA).

## RESULTS

### *In vitro* interaction of glycosaminoglycans with α-synuclein and cathepsin D

**Figure 1:**
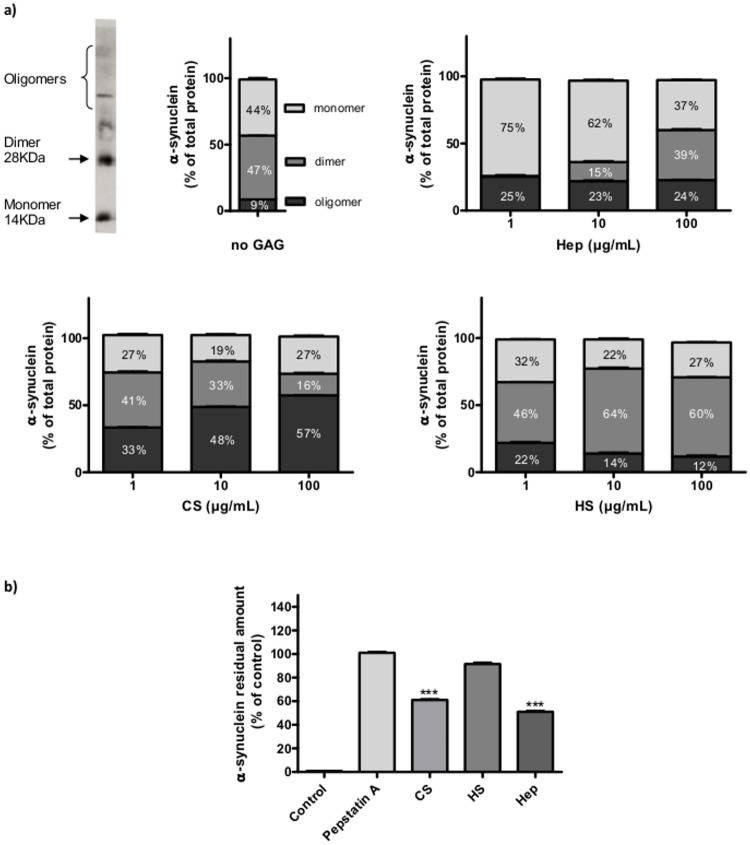
Glycosaminoglycans modulation of α-synuclein aggregation and degradation by cathepsin D *in vitro*. a) *In vitro* α-synuclein aggregation in the presence of different GAGs. Commercial α-synuclein (25 μg/mL) was incubated 24 h at 37°C in the presence of 1, 10 or 100 μg/mL of commercial Hep, CS or HS. Monomeric, dimeric and oligomeric forms of α-synuclein were detected by western blot and quantified with ImageJ software. b)Inhibition of cathD degradation of α-synuclein by different commercial GAGs. α-synuclein was incubated in the presence of cathD (50 mU/mL) and pepstatin A, Hep, HS or CS (100 μg/mL) during 30 min at 37°C. Residual α-synuclein was detected by western blot and quantified with ImageJ software. Results are presented as the mean ± S.E.M. *** p<0.01 compared to control.

**Glycosaminoglycans modulation of α-synuclein oligomerisation in vitro**. The ability of GAGs to increase oligomerisation of α-synuclein *in vitro* was investigated using commercial GAGs (Fig. 1a). After 24 h of incubation, α-synuclein was detected as monomers (9±1.1%), dimers (47±3.5%) and oligomers (44±2.4%). In the presence of 1 μg/mL of Hep, most of the α-synuclein was monomeric (75%) and 25%, oligomeric. In contrast, co-incubation of α-synuclein with HS or CS did not increase the proportion of dimers, while monomers represented only 25% and oligomers 20 to 35% of α-synuclein forms. Increasing Hep concentrations (10 and 100 μg/mL) rose the percentage of α-synuclein dimers, whereas the oligomers formation was greater at higher CS concentrations. Interestingly, in the presence of 10 and 100 μg/mL HS, the percentage of dimers and monomers was amplified whereas the oligomeric forms of α-synuclein tended to decrease.

**Glycosaminoglycans modulation of α-synuclein degradation by cathepsin D *in vitro***. *In vitro*, cathD degraded recombinant α-synuclein in a dose-dependent manner with an EC_50_ of 1.97 mU/mL (S1 Fig.) whereas this degradation activity was totally inhibited by its specific inhibitor pepstatin A (peps, 0.3mM, Fig. 1b). Interestingly, the *in vitro* degradation of α-synuclein by cathD was inhibited by 100μg/mL Hep and CS but not by HS. These results suggest that GAGs, depending on their chemical structure, differentially regulate cathD activity.

### Cellular model characterization

**Figure 2:**
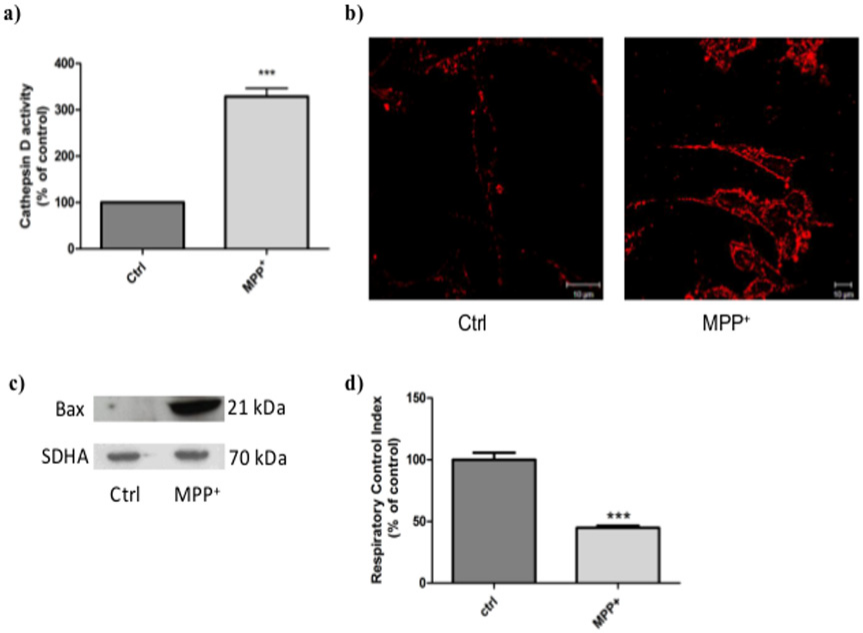
Characterization of MPP^+^-induced apoptosis. Total, mitochondrial and cytosolic extracts were obtained from normal and MPP^+^-stressed SHSY5Y cells (0.5 mM, 6 h).a) CathD activity in cytosolic extracts. CathD activity was normalized by cell number in each sample and expressed as percentage of the activity in normal cells. Results are expressed in percentage of normal cell extract levels and presented as mean ± S.E.M. of four independent experiments in triplicate. *** p<0.01 compared to control cells. b) Immunofluorescence detection of total cathD in cells. Left: unstressed control cells (Ctrl), right: MPP^+^-stressed cells (6 h). Cells were observed with a confocal microscope Zeiss Axio Observer Z.1. Scale bar represents 10 μm. Negative control omitting the first antibody but in the presence of the second one (anti-goat Fluo 546) did not show any signal. c) Analysis by western blot of Bax relocation in mitochondrial membranes at 6 h, just after the end of stress. The amount of protein was normalized by the number of cells to avoid taking into account the increase of protein amount induced by the MPP^+^ stress. Succinate dehydrogenase-A (SDHA) was used as the loading control. This immunoblot is representative of three independent experiments in duplicate. d) Assessment of respiratory chain function 24 h after MPP^+^ treatment. The respiratory control index (RCI) i.e. ratio [state 3 rate] / [state 4 rate] was calculated. Results are expressed as percentage of unstressed control group and represent three independent experiments in triplicate. Results are presented as the mean ± S.E.M. *** p<0.01 compared to control cells.

**MPP^+^ activation of the intrinsic apoptosis pathway.**We investigated the MPP^+^-induced apoptosis pathway, in particular the pre-mitochondrial events like the release of cathD from lysosomes, which is poorly documented. Results in Fig. 2a showed a 3-fold increase of cathD activity in cytosolic extracts from MPP^+^-stressed cells. This observation was correlated with the localization of cathD by confocal microscopy (Fig. 2b) in MPP^+^-treated cells compared to unstressed control cells. In MPP^+^-stressed cells, cathD seemed to be more present in the cytosolic compartment whereas in control cells, staining appeared more punctuated, consistent with the lysosomal storage of the protease. CathD release into the cytosol leads, by an unknown mechanism, to Bax activation and to its relocation into mitochondria, which mediates apoptosis [19, 31]. Bax relocation in the mitochondrial membrane was detected by western blot at the end of the stress period induced by MPP^+^ treatment (Fig. 2c). The significant increase of Bax in the mitochondrial membrane of stressed cells indicated that MPP^+^ exposure effectively induced Bax activation, leading to a 50% inhibition of the respiratory control index compared to unstressed control cells (Fig. 2d). These events are typical markers of the intrinsic pathway of apoptosis that were confirmed by caspase-9 and -3 activation (S2 Fig.).

**Figure 3:**
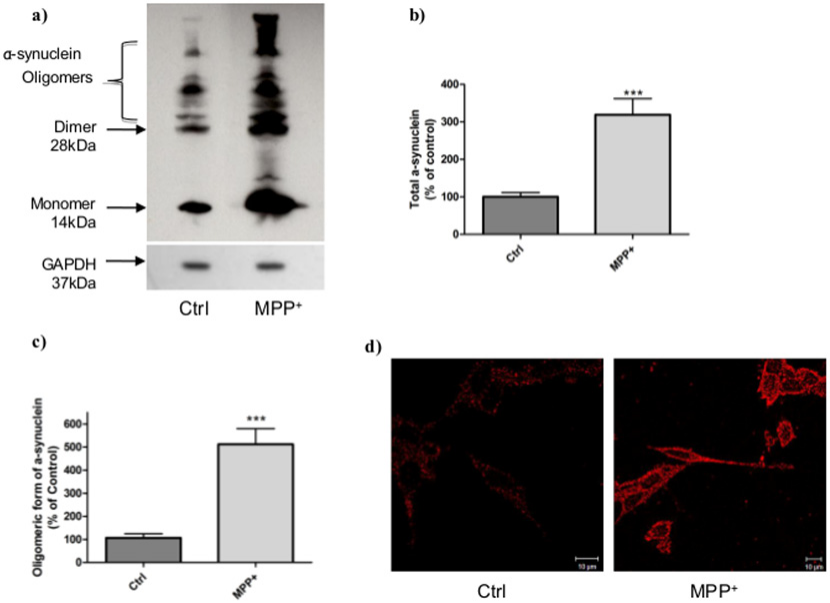
Characterization of α-synuclein aggregation in cells after MPP^+^ stress. a) Typical immunoblot of α-synuclein in stressed (MPP^+^, 6h)) and unstressed (Ctrl) cells. b) Total and c) oligomeric forms of α-synuclein measured on stressed (MPP^+^) and unstressed (Ctrl) cells by Western Blot and quantified by ImageJ software. This immunoblot represents three independent experiments and is expressed as the mean ± S.E.M. *** p<0.01 compared to control cells. GAPDH was used as loading control. d) α-synuclein aggregation immunostaining with α-synuclein antibody in unstressed cells (Ctrl) and MPP^+^-stressed cells. Cells were observed with a confocal microscope Zeiss Axio Observer Z.1.

**MPP^+^ induction of intracellular α-synuclein accumulation.**To determine whether MPP^+^ treatment could induce other modifications, we compared α-synuclein levels by Western Blot (Fig. 3a). A significant 3-fold increase in total α-synuclein (Fig. 3b) and 5-fold increase in oligomeric forms were observed in stressed cells compared to control cells (Fig. 3c). Intracellular α-synuclein, assessed by immunostaining (Fig. 3d), was also modified in control *vs*. stressed cells. Indeed, in control cells, α-synuclein was localized in punctuated forms distributed in the cytosol whereas in stressed cells, the protein was clearly more expressed and everywhere at the intracellular level.

**Consequences of MPP^+^ stress on glycosaminoglycans and their biosynthesis.**In order to test whether MPP^+^ affected GAGs amounts, species and structure in our stress cell model, GAGs were extracted and characterized. Total GAGs and HS/CS amounts were quantified by using selective enzymatic treatments and the cationic dye DMMB assay [27, 28]. After treatment by MPP^+^, total sulfated GAGs raised to 1.85±0.27 μg/10^6^cells (Table 1) compared to 1.26±0.14 μg/10^6^cells in control unstressed cells, corresponding to 1.5 fold increase. Among these, HS increased from 55 to 67%, whereas CS decreased from 45 to 33% in MPP^+^-stressed cells (Table 1). Moreover, the disaccharide composition of HS and CS was modified by MPP^+^ treatment. In HS extracted from MPP^+^-stressed cells, the percentage of non-sulfated GAGs significantly decreased by 8%, whereas the proportion of Mono-D and Di-S increased. In CS, the non-sulfated disaccharides decreased significantly from 58.3±6.9% to 39.1±5.5%, while Mono, Di and Tri-sulfated disaccharides percentages were increased.

**Table 1:** Glycosaminoglycans in control and MPP^+^-stressed cells.

	**Total GAGs (μg/10^6^cells)**	**HS/CS (% of total GAGs)**		**Disaccharides (% of total amount)**			
				**Non-Sulfated**	**MonoSulfated**	**DiSulfated**	**TriSulfated**
Control	1.26±0.14	HS	55±4.7	89.15±5.31	-	9.15±4.50	-
		CS	45±3.8	58.33±6.99	8.72±1.44	29.50±3.88	3.20±2.00
MPP^+^	1.85±0.27**	HS	67±5.1*	81.10±5.32*	1.57±0.54	14.13±4.71*	0.08±0.02
		CS	33±4.0*	39.13±5.56**	12.30±2.39*	43.45±1.04**	5.10±2.10

*p<0.01,

**p<0.05 compared to control cells. Results are the mean ± S.E.M. of three independent experiments in duplicate.

**Figure 4:**
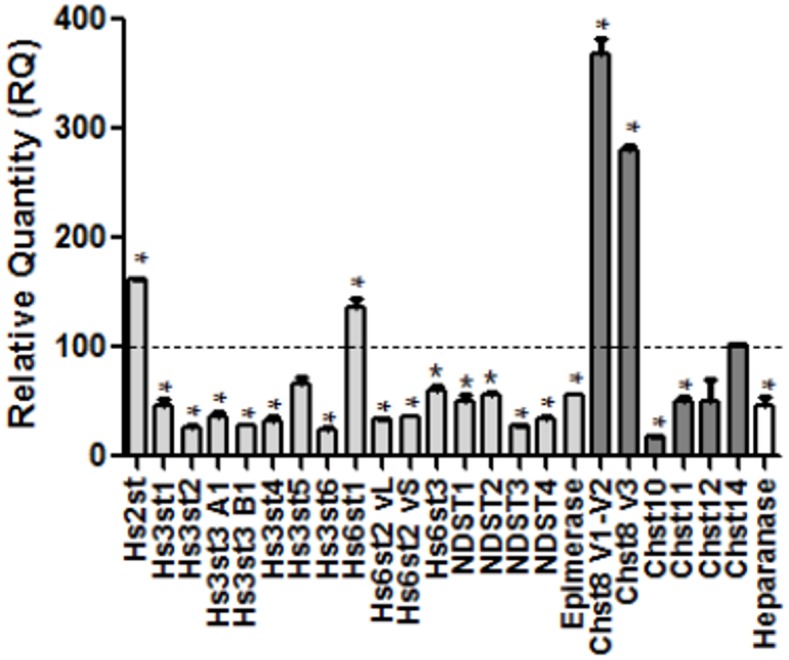
GAGs metabolism in normal and MPP^+^-stressed cells. Analysis of enzymes involved in GAGs metabolism by Real Time PCR. Real time PCR of Hs2st, Hs3st1, Hs3st2, Hs3st3, Hs3st3A1, Hs3st3B1, Hs3st4, Hs3st5, Hs3st6, Hs6st1, Hs6st2vL, Hs6st2vS, Hs6st3, NDTS1, NDST2, NDST3, NDST4, Chst8 v1 and v2, Chst8 v3, Chst10, Chst11, Chst12, Chst14, epimerase and heparanase were performed with RNA extracts from MPP^+^-stressed (24 h) and normal cells. Values were normalized using geNorm software, an accurate normalisation of gene expression with multiple references genes [30]. The following reference genes glyceraldehyde 3-phosphate dehydrogenase (GAPDH), glutamine synthetase and alpha-tubulin were used. Each measure is the mean +/- S.E.M. from measurements performed on 3 independent cultures. * p<0.01 compared to control cells. Light grey bars: HS biosynthesis enzymes; dark grey bars: CS biosynthesis enzymes; white bar: HS degradation enzyme.

In the same conditions of stress, the gene expression of enzymes involved in GAGs biosynthesis was studied (Fig. 4). Among the 24 genes screened, only Hs2st, Hs6st1, Chst8 and Chst8 var3, were significantly increased compared to control cells whereas Hs3st5, Chst12 and Chst14 remained unchanged. The expression levels of all the other genes encoding GAGs biosynthetic enzymes were decreased by less than 2 fold, except Hs3st6 and Chst10, which expression decreased over 4-fold. Interestingly, the expression level of HS degrading enzyme, heparanase, was also decreased by 2 fold. Thus, the stress induced by MPP^+^ modified both HS and CS structure and quantity, partly through the regulation of GAGs biosynthetic enzymes expression.

### Glycosaminoglycans roles in MPP^+^ cellular model

**Figure 5:**
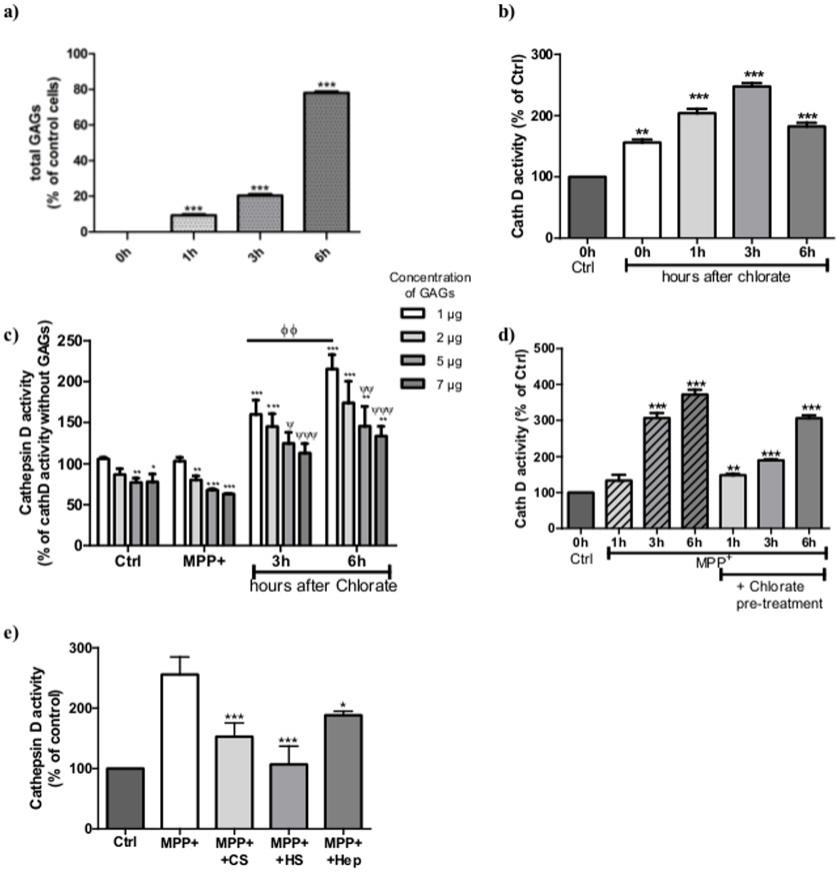
Regulation of cathepsin D activity by glycosaminoglycans. a) SH-SY5Y cells were treated with sodium chlorate (75 mM) during 24 h and sample were extracted at different times after sodium chlorate removal. GAGs amounts were measured in cells after 0, 1, 3 and 6 h after the end of the chlorate treatment. Results are presented as mean ± S.E.M. of three independent experiments in triplicate. *** p<0.001 compared to control. b) Total cathD activity was measured at different times after sodium chlorate removal (75 mM, 24h). Results are expressed in percentage of cathD in normal cell extract and presented as mean ± S.E.M. of three independent experiments in triplicate. *** p<0.001 compared to control cells. c) GAGs were extracted from control, MPP^+^-stressed cells, chlorate treated cells 3 and 6 h after chlorate removalas described in Material and Methods. GAG amounts were measured by DMMB assay [27]. The activity of cathD was measured in the presence of various concentrations of these extracted GAGs (white bars: 1μg/mL; light grey2μg/mL, medium grey:5 μg/mL and dark grey: 7 μg/mL) and expressed as the percentage of the specific CathD activity. Results are mean ± S.E.M. of three independent experiments in duplicate. ** p<0.005; *** p<0.001 compared to control cathD activity, ψ p<0.01; ψψ p<0.005; ψψψ p<0.001 compared to results obtained with 1 μg/mL of GAGs, ΦΦ p<0.005 comparison between 3 and 6 h extracted GAGs. d) Total cathD activity was measured after different times (0, 1, 3 and 6 h) of treatment with MPP^+^ (0.5 mM) with or without pre-treatement of cell with chlorate (75 mM, 24h). CathD activity was normalized by cell number in each sample and expressed as percentage of the activity in normal cells. ** p<0.005; *** p<0.001 compared to control. e) CathD activity in cells subjected or not to MPP^+^ stress (6 h) and followed by commercial GAGs treatment (Hep, HS and CS, 1 μg/mL). After 24 h, GAGs effects were compared to the complete inhibition of cathepsin activity by pepstatin A (peps), a specific inhibitor of this enzyme and expressed as percentage of cathD activity in control cells.

**Regulation of cathepsin D activity by glycosaminoglycans**. At the end of sodium chlorate treatment (0 h, Fig. 5a), by which cell viability was shown unaffected, the absence of sulfated GAGs obtained by the inhibition of the sulfate donor PAPS was confirmed by quantification using the DMMB test [22]. Thus, neo-synthesis of sulfated GAGs occurred and their amounts showed a remarkable 4-fold increase, from 20% at 3 h reaching to 80% of the normal amount in control cells after 6 h (Fig. 5a). The sodium chlorate treatment did not induce caspase-9 and -3 activations, but increased total cathD activity in cells from 160% at 0 h, to 250% at 3 h, which then decreased to 180% after 6 h (Fig. 5b). The ability of GAGs extracted from cells to inhibit cathD activity was investigated in various experimental conditions, i.e. control, MPP^+^-stressed and 3 or 6 h after sodium chlorate treatment (Fig. 5c). CathD activity was not significantly modified by the presence of GAGs extracted from stressed and unstressed cells at doses up to 1 μg/mL in the reaction media. However, at higher amounts of GAGs, an important decrease of cathD activity was induced, from 19–23% at 2 μg/mL reaching 28–35% at 7 μg/mL (Fig. 5c). Surprisingly, 1 μg/mL of GAGs extracted after chlorate withdrawal, induced a dramatic 1.3-fold increase of cathD activity between 3 and 6 h. The increase in concentration of these particular GAGs induced a dose-dependent reduction of cathD activity, reaching 113% and 133% at 7 μg/mL of GAGs at 3 and 6 h, respectively (Fig. 5d). This puts forward for consideration a fine structure activity relationship, where both sulfation and concentration of these sulfated GAGs, seem to play an important role. Cell treatment with MPP^+^ increased time-dependently intracellular cathD activity, with a maximum effect of 372% at 6 h (Fig. 5d). Pre-treatment of cells by sodium chlorate before MPP^+^ exposure, maintained cathD activity at low levels for up to 3 h, whereas an important increase (306%) was observed at 6 h.

In order to evaluate the effect of the sulfation level and patterns, we tested the effect of cell exposure to different natural GAGs after MPP^+^stress on cellular cytosolic cathD (Fig. 5e). The increase of cathD activity induced by MPP^+^ can be efficiently inhibited by cell treatment with HS, and to a lesser extent by CS and Hep. Taken together, these data put forward the importance of the intracellular sulfated GAGs for the regulation of cathD activity.

**Figure 6:**
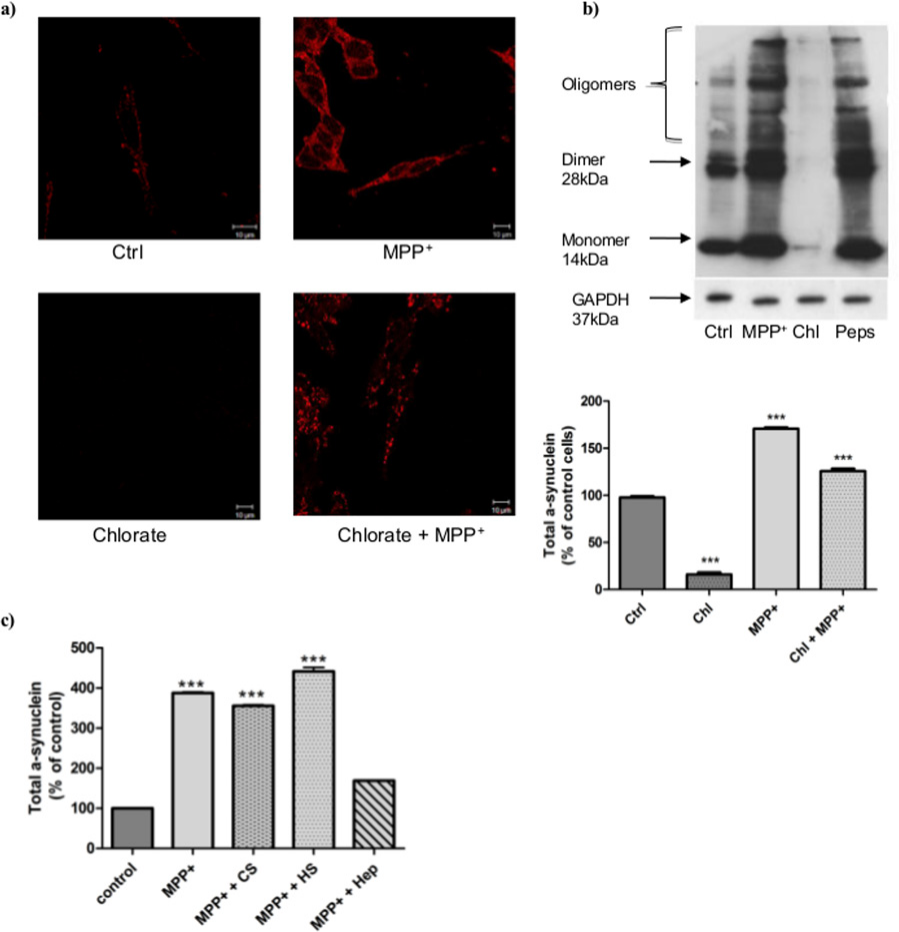
Effect of glycosaminoglycans sulfation on α-synuclein accumulation. a) Total α-synuclein accumulation was detected after 6 h in normal (Ctrl) or MPP^+^-treated cells and after chlorate treatment (75 mM, 24h). α-synuclein aggregation was visualized by immunostaining. Cells were observed with a confocal microscope Zeiss Axio Observer Z.1. b) Western blot analysis of total α-synuclein in lysate of cells subjected or not to MPP^+^ stress (6 h) after treatment with sodium chlorate (75 mM, 24h) or the specific inhibitor of cathD, pepstatin A (Peps) (100 μM, 24h). Intensities of the bands obtained by western blot were quantified by ImageJ software and represent three independent experiments and are expressed as the mean ± S.E.M. *** p<0.001 compared to control cells. GAPDH was used as the loading control. c) Amounts of total α-synuclein in cells subjected or not to MPP^+^ stress (6 h) and followed by commercial GAGs treatment (Hep, HS and CS, 1 μg/mL). After 24 h, α-synuclein was detected by western blot and quantified with ImageJ software.

**Importance of glycosaminoglycans sulfation on α-synuclein accumulation**. As exogenously added GAGs modulated CathD activity in MPP^+^-stressed cells (Fig. 5e), we examined the importance of GAGs sulfation on α-synuclein accumulation. In sodium chlorate-treated cells, α-synuclein was almost completely undetectable by immunostaining and western blot (Fig. 6a–b). In contrast, 6 h of MPP^+^ stress induced an important increase of intracellular α-synuclein as seen by western blot (170%), and by immunostaining. Pre-treatment with sodium chlorate before MPP^+^ stress caused a significant reduction of α-synuclein amount (from 170 to 130%) compared with MPP^+^-treated cells, without restoring the level of unstressed cells. Interestingly, the inhibition of endogenous cathD activity by pepstatin A prevented α-synuclein degradation (Fig. 6b) leading to dimers and oligomers amounts close to those observed with the MPP^+^treatment. Moreover, this α-synuclein accumulation induced by MPP^+^ was increased 4 fold (400%) after 24 h, but could be reduced to 174% by Hep treatment just after MPP^+^-stressed period (Fig. 6c). In these experimental conditions, HS and CS were unable to interfere on the increase of α-synuclein induced by MPP^+^, whereas all these GAGs had no effect on α-synuclein in normal unstressed cells.

**Co-localization of cathepsin D, glycosaminoglycans and α-synuclein in MPP^+^-stressed cells.**As regulation of cathD by GAGs through their sulfation pattern was suggested to modulate α-synuclein accumulation/degradation (Figs. 2, 5, 6) we investigated whether they could co-localize in cells.

**Figure 7:**
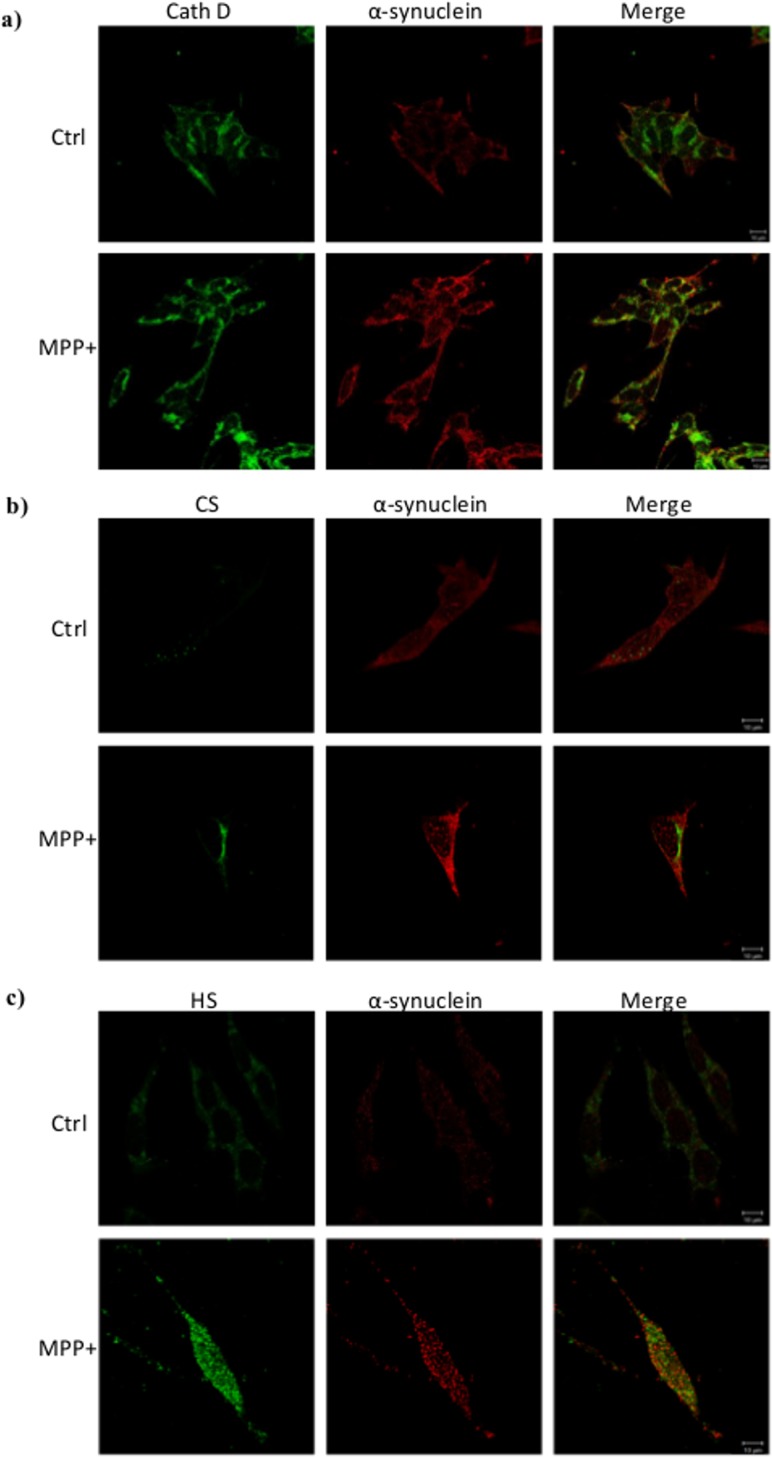
Co-localization of cathepsin D, glycosaminoglycans and α-synuclein in MPP^+^-stressed cells. a) Immunofluorescence detection of CathD (green) and α-synuclein (red) in unstressed (Ctrl) and MPP^+^-stressed cells (6 h). b) Immunofluorescence co-labeling of CS (green) and α-synuclein (red) in normal (Ctrl) and MPP^+^-stressed cells (6 h). c) Immunofluorescence co-labeling of HS (green) and α-synuclein (red) in normal (Ctrl) and MPP^+^-stressed cells (6 h). Observations were done with a confocal microscope Zeiss Axio Observer Z.1. Stars and arrows indicate areas where co-localization is observed.

Whereas the double labeling showed distinct localizations for cathD and α-synuclein in normal cells (Fig. 7a), in contrast, cathD and α-synuclein partly co-localized in MPP^+^-stressed cells. Concerning GAGs, the double labeling revealed that cathD did not co-localize with HS and CS in normal cells, whereas in MPP^+^-treated cells, in which the amounts of both GAGs and cathD were enhanced, an important co-localization appeared (S3 Fig.). Interestingly, the poor labeling of CS in normal cells and the peri-nuclear location of CS in MPP^+^-stressed-cells, did not colocalize with α-synuclein whatever the cell status (Fig. 7b). By contrast, HS only colocalized with α-synuclein in MPP^+^-stressed cells (Fig. 7c), suggesting the presence of HS in the cytosol and the existence of a complex HS- α-synuclein.

## DISCUSSION

Substantial amount of evidence suggests that the oligomerisation of α-synuclein followed by its aggregation is a critical step in the etiology of PD [32, 33]. However, if one of the hallmarks of PD is the presence of LB, mainly constituted of α-synuclein, the mechanism underlying their formation and their pathogenic relevance remain unclear. Few studies have suggested the presence of GAGs in LB, where they can interact with α-synuclein, notably at an early stage of aggregation, prior to fibrillation [34]. With the aim of studying early stage of PD, characterized by the presence of mono-, di- and oligomeric α-synuclein forms, we used a semi-quantitative western-blot method, able to detect very low amount of α-synucleinoligomers compared to other classical methods [35]. *In vitro*, using this method, we confirmed that GAGs can modulate the α-synuclein transition between these forms that are resistant to SDS exposure during Western-blot. *In vitro*, increasing concentrations of Hep and HS, to a lesser extent, both augment the proportion of dimers, whereas CS induces the increase of oligomers. Thus, the *in vitro* ability of α-synuclein to form dimers/oligomers, appears to be differently regulated by GAGs compositions and concentrations. *In vivo*, oligomerisation and aggregation of α-synuclein are more complex, probably involving unknown enhancers/processes of aggregation and an impairment of its clearance that may be caused by alterations in the UPS or the ALP [9]. Recently, studies suggested that the UPS is the main degradation pathway for α-synuclein under normal conditions, while the ALP is recruited when the α-synuclein expression is increased [11]. Among the most powerful hydrolytic enzymes in lysosomes is cathD, a major intracellular aspartic protease, described as the main α-synuclein degrading enzyme [14].

*In vivo*, cathD is classically involved in apoptosis through its release from lysosome and its activation of the pro-apoptotic protein Bax in the cytosol. In this study, we used the gold standard of toxin-based PD models by treating neuroblastoma cells with the MPTP metabolite MPP^+^, a potent mitochondrial complex I inhibitor. For the first time, we demonstrate that a time-dependent increase of cathD activity and expression occurs in MPP^+^-stressed cells. A 6 hour MPP^+^ treatment is sufficient to activate the intrinsic pathway of apoptosis characterized by cytosolic cathD release, Bax translocation and caspase-9 and -3 activation. The mitochondrial dysfunction observed after MPP^+^ treatment, with the decrease of the respiratory control index (RCI), also suggests the inhibition of complex I and the overproduction of reactive oxygen species [36] leading to lysosomal membrane disruption and release of cathD [19]. This increase of lysosomal membrane permeabilization, resulting in the collapse of lysosomal function, was suggested to end up with the impairment of cytoprotective autophagy and neuronal cell death in a model of PD [37]. In the cytosol, cathD activates Bax by an unknown mechanism that can induce, in a vicious circle, the collapse of mitochondrial membrane potential and the release of cytochrome c. Similarly, the up-regulation of cathD recently observed in the caudate nucleus of rhesus monkeys treated chronically by MPTP, correlated with cellular damages leading to typical caspases activation of apoptosis [17] and thus corroborates our results. Remarkably, we observed the concomitant and significant increase of α-synuclein accumulation at the intracellular level. This highlights the discrepancy between the presence of cathD and its inefficiency to prevent α-synuclein accumulation, and suggests the existence of a fine control of the protease activity by endogenous systems, including natural inhibitors. Interestingly, western-blotting assay of α-synuclein was performed in the presence of SDS, which cannot dissociate oligomers of α-synuclein as previously described by Tsika et al [38]. Furthermore, the molecular weight bands patterns corresponding to dimers, trimers, and multimers of α-synuclein and resistant to SDS detected in our cellular study, were similar to those described in α-synuclein sample extracted from different mouse brain areas [38]. This strengthens the interest of our cellular model induced by MPP^+^ cell exposure to study the regulation of α-synuclein accumulation and early stage of oligomerisation processes.

We and others have shown the potential of GAGs, present within the lysosomes for degrading processes, to inhibit cathD activity [19, 39]. Thus, we investigated modifications of GAGs in our cellular MPP^+^ model and found an increase of total GAGs in MPP^+^-stressed cells, associated to an increase of HS part. This finding can be linked to the decreased expression of heparanase, one of the main GAGs degrading enzymes that we previously described to be unchanged in hippocampus during ageing [29]. Remarkably, the impairment of HS degradation was recently suggested to play a role in α-synuclein accumulation in PD due to the mutation in NAGLU gene (rs2071046), which typically leads to the accumulation of HS in lysosomes in Sanfilippo syndrome, a human storage disorder [21]. Considering both our results and those of Winder-Rhodes [21], GAGs, for the first time, could be hypothesized as major players in the regulation of cathD activity and α-synuclein aggregation. Furthermore, the up-regulation of the expression of Hs2st1 and Hs6st1, two genes involved in the first steps of sulfation in HS biosynthesis and HS disaccharide analysis after MPP^+^ stress period, whereas all the others genes were down-regulated, clearly supports the modification of the sulfation patterns in HS. Indeed, Hs2st1 transfers the sulfo group to the 2-OH position of iduronic acid (IdoA) or glucuronic acid (GlcA) within HS, whereas Hs6st1 catalyzes a 6-*O* sulfation of *N*-acetylglucosamine (GlcNAc). Interestingly, the expression of these two genes that was shown to be regulated by oxidative stress in a skin keratinocyte cell line [40], correlated with the increase of Di and Tri-sulfated HS-disaccharides measured after MPP^+^ exposure. Thus, the increase of HS amount can be associated with a modification of HS sulfation pattern.

In contrast, after MPP^+^ stress, enzymes of CS biosynthesis were down-regulated, correlating with CS amount measurement, except Chst8 that was over-expressed suggesting a modification of sulfation pattern reinforced by the variation of Mono, Di and Tri-sulfated disaccharides measured in MPP^+^-stressed extracted GAGs. The full-length Chst8 protein classically has a GalNAc-4-sulfotransferase activity and transfers a sulfate group to the carbon 4 of *N*-acetylgalactosamine (GalNAc) residues of *N*-glycoproteins, whereas the truncated form of Chst8 does so to the carbon 4 of GalNAc residues of CS chains [41]. This could be of importance if we consider that these 4-sulfated CS-A, mainly present in adulthood, [42] are described to inhibit axon regeneration [43]. Moreover, the down-regulation of this 4-*O*-sulfation was suggested to be linked with PrP(sc) accumulation in Creutzfeldt-Jacob disease [44]. However, in our experiment, extracted GAGs from both normal and MPP^+^-stressed cells did not modify cathD activity *in vitro* when they were used at 1 μg/mL (although the nature of these GAGs is likely different), whereas at higher concentrations, they inhibited cathD activity. Noteworthily, neo-synthesized GAGs after complete unsulfation with sodium chlorate treatment, markedly increased cathD activity at 1 μg/mL, while higher concentrations of GAGs were less efficient to stimulate cathD activity. Consequently, the increase of GAGs amount induced by MPP^+^ stress or the neosynthesis of GAGs after chlorate treatment suggest a complex and sophisticated balance between the existence of specific sulfation pattern in GAGs and the importance of intracellular GAGs amount to explain their specificity of effects at the intracellular level.

We recently described heparan sulfate proteoglycans as critical mediators of cell uptake by macropinocytosis and seeding of tau aggregate and α-synuclein fibrils [22]. The mature macropinosomes containing both heparan sulfate proteoglycan, i.e. GAGs, and α-synuclein, were degraded afterwards in the late endosomes lysosomes. Thus, the presence of GAGs and cathD together in lysosomes supports the hypothesis that cathD activity and α-synuclein accumulation could be regulated by GAGs. This idea is reinforced by the recent discovery of mutations in the NAGLU gene in Sanfilippo syndrome and PD [21], resulting in the accumulation of partial HS chains within the lysosomes as a consequence of the disruption of their degradation. Remarkably, in our cellular model, sulfated GAGs amount dramatically increased after MPP^+^ stress, probably, and partly, through the inhibition of heparanase expression. The abolition of sulfation in GAGs increased cathD activity and abolished almost completely intracellular α-synuclein amount, which reinforces the potential regulation of these events by GAGs. The colocalization studies in MPP^+^-stressed cells suggest a potential association of GAGs with cathD or α-synuclein, resulting in α-synuclein detection, whereas in the absence of GAGs/cathD co-localization i.e. in normal cells, α-synuclein is not detected, probably due to its degradation by cathD. Moreover, the co-labelling of HS with α-synuclein on the one hand, the co-localization of α-synuclein and CathD on the other hand, and the increase of CathD activity in cytosolic extract of MPP^+^-stressed-cells suggests the presence of HS in cytosolic compartment associated with cathD and α-synuclein.

Finally, treatment of cells with commercial GAGs after MPP^+^ stress suggests that extracellular GAGs can enter inside the cells to differentially modulate, depending on their structure, i.e. mainly sulfation patterns, both α-synuclein accumulation in lysosomes and pro-apoptotic cathD activity in cytosol. These results are of interest to understand the pathological processes of spreading in synucleinopathies, considering the role of GAGs, mainly HS, in cell uptake of α-synuclein [22]. MPP^+^ exposure increasing the sulfation of HS, which delays pro-apoptotic activity of cathD and reduces the α-synuclein degradation, leads to an increase of α-synuclein accumulation and facilitates the spreading from cell to cell.

**Figure 8:**
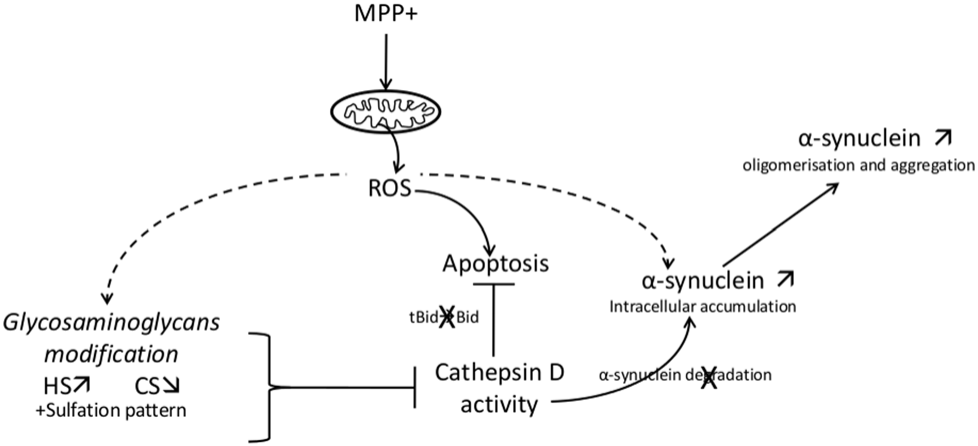
Interplay between CathD, glycosaminoglycans (heparan sulfate (HS), chondroitin sulfate (CS)) and α-synuclein on MPP^+^-stressed cells. See details in the text.

In conclusion, this study describes cathD and GAGs as new molecular actors in MPP^+^ toxicity. Furthermore, GAGs are proposed for the first time, as new and major actors in the lysosome degradation pathway, by regulating cathD activity and affecting two main biological processes, protein aggregation/degradation—in our case α-synuclein—and apoptosis (Fig. 8). These findings strengthen the interest on the regulation of lysosomal biogenesis and functions occurring in neurodegenerative disorders, particularly those with α-synuclein accumulation and aggregation, or with GAGs degradation and storage impairment [21]. Indeed, the demonstration that cathD activity is regulated by endogenous GAGs and the consequences of this regulation on α-synuclein accumulation highlight the importance of glycobiology in neurodegenerative disorders such as PD, and potential developments of new therapeutic strategies.

## Supporting Information

S1 Table Primers sequences used in Real time RT-PCR. (TIF)

S1 Fig α-synuclein degradation by cathepsin D. α-synuclein degradation *in vitro* in the presence of different concentrations of cathD during 30 min at 37°C. The residual amount of α-synuclein was detected by western blot and quantified with ImageJ software. Results were represented as percentage of control (100% was obtained in the presence of pepstatin A (peps), a specific inhibitor of cathD). Results are presented as the mean ± S.E.M.
(TIF)

S2 Fig Caspase-3 and -9 activities. Caspase-9 and -3 activities were measured at 6 and 24 h, respectively after the end of MPP^+^ treatment, using a specific fluorescent substrate, Ac-LEHD-AFC for caspase-9 (a) and Ac-DEVD-AFC as substrate for caspase-3 (b). Results are expressed as percentage of unstressed control group and represent three independent experiments in triplicate. Results are the mean ± S.E.M. *** p<0.01 compared to control cells. Cells treated under various conditions were harvested through trypsinization and washed with PBS. The cell pellet was gently suspended in buffer containing 30 mM HEPES, 0.3 mM EDTA, 100 mM NaCl, 0.15% Triton X-100 and 10 mM DTT and centrifuged. The supernatant was used for the assay. Caspases substrates were added to a final concentration of 100 mM. The plate was covered, gently mixed and incubated at 37°C for 1 h. Then samples were measured at λ_ex_ 400 nm and λ_em_ 505 nm in a fluorescent microplate reader (TECAN infinite M1000).
(TIF)

S3 Fig Co-localization of cathepsin D and glycosaminoglycans in MPP^+^-stressed cells. Immunofluorescence co-labeling of cathD (green) and endogenous HS-CS (red) in normal (Ctrl) and MPP^+^-stressed cells (6 h). Observations were done with a confocal microscope Zeiss Axio Observer Z.1. Stars and arrows indicate areas where co-localization is observed.
(TIF)
