# Supplementary figures and images for: New Roles of Glycosaminoglycans in α-Synuclein Aggregation in a Cellular Model of Parkinson Disease
Source: PLoS One. 2015 Jan 24;10(1):e0116641. doi: 10.1371/journal.pone.0116641 (PMC4305359; doi:10.1371/journal.pone.0116641)

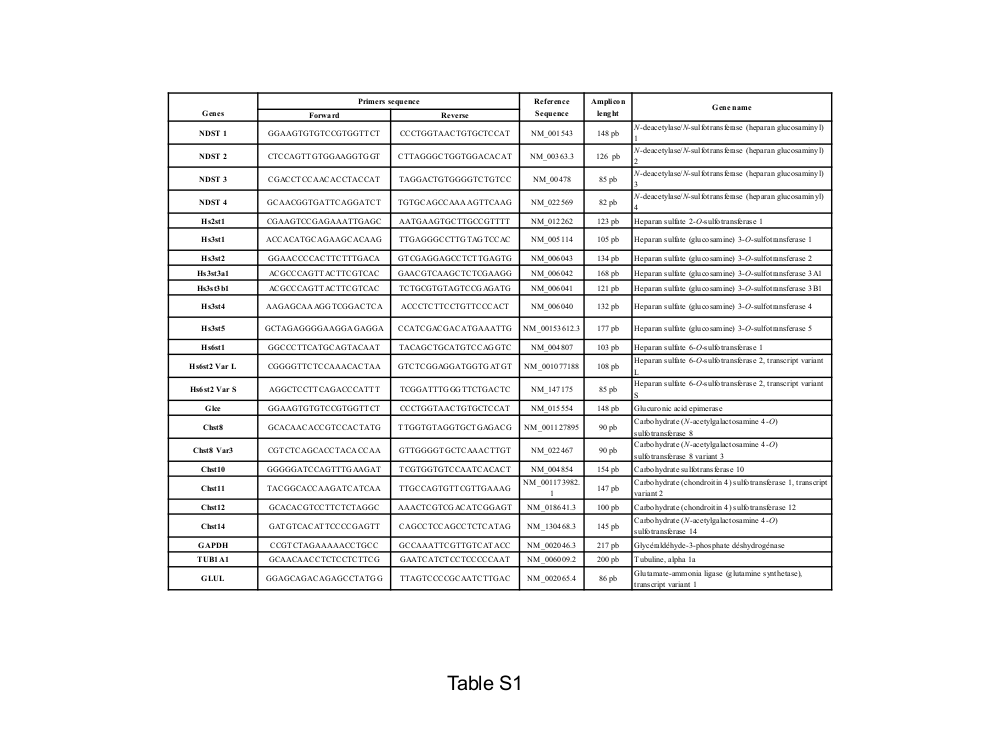

Supplement: S1 Table — (TIF) [file pone.0116641.s001.tif]

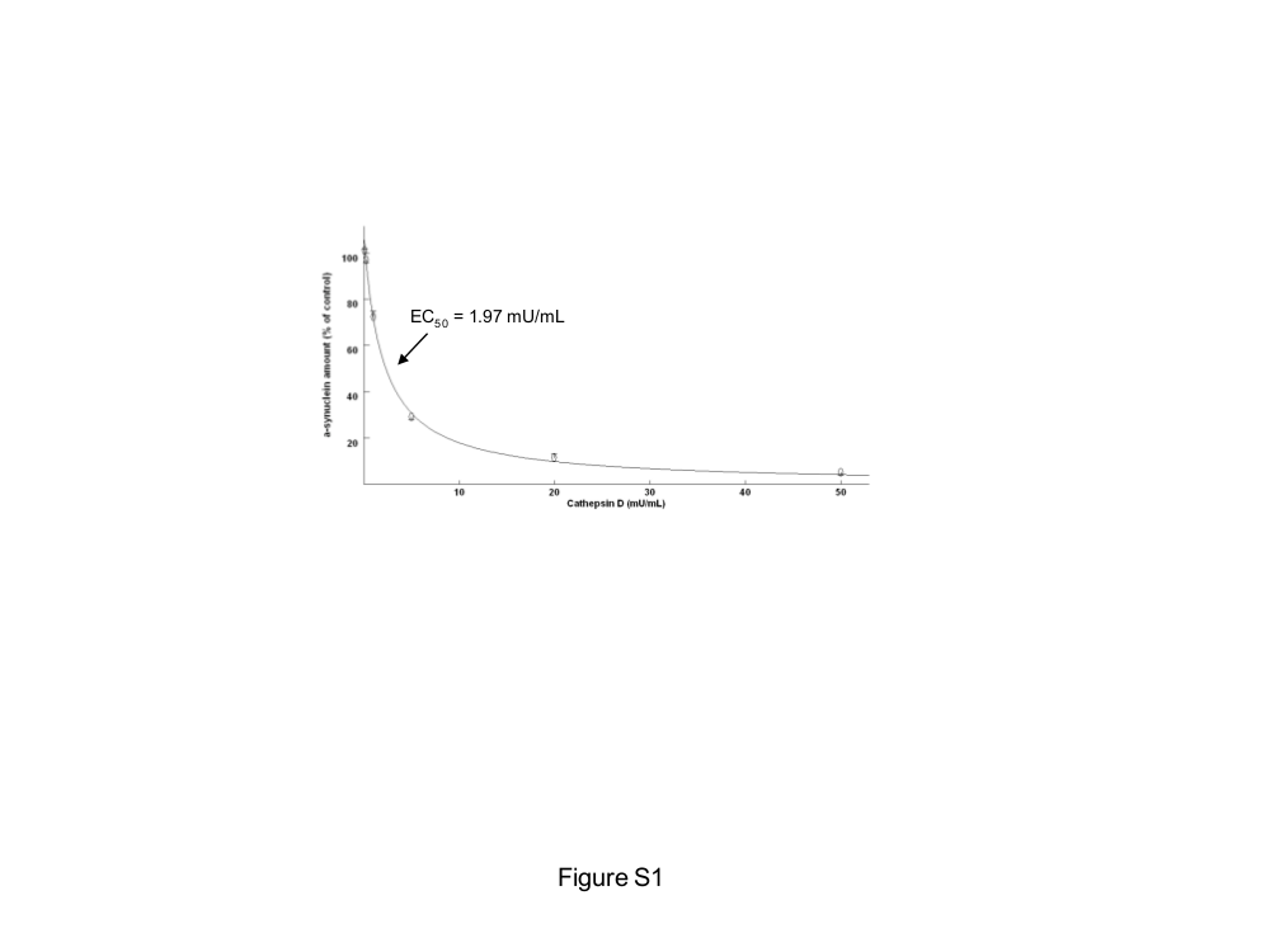

Supplement: S1 Fig — α-synuclein degradation in vitro in the presence of different concentrations of cathD during 30 min at 37°C. The residual amount of α-synuclein was detected by western blot and quantified with ImageJ software. Results were represented as percentage of control (100% was obtained in the presence of pepstatin A (peps), a specific inhibitor of cathD). Results are presented as the mean ± S.E.M. (TIF) [file pone.0116641.s002.tif]

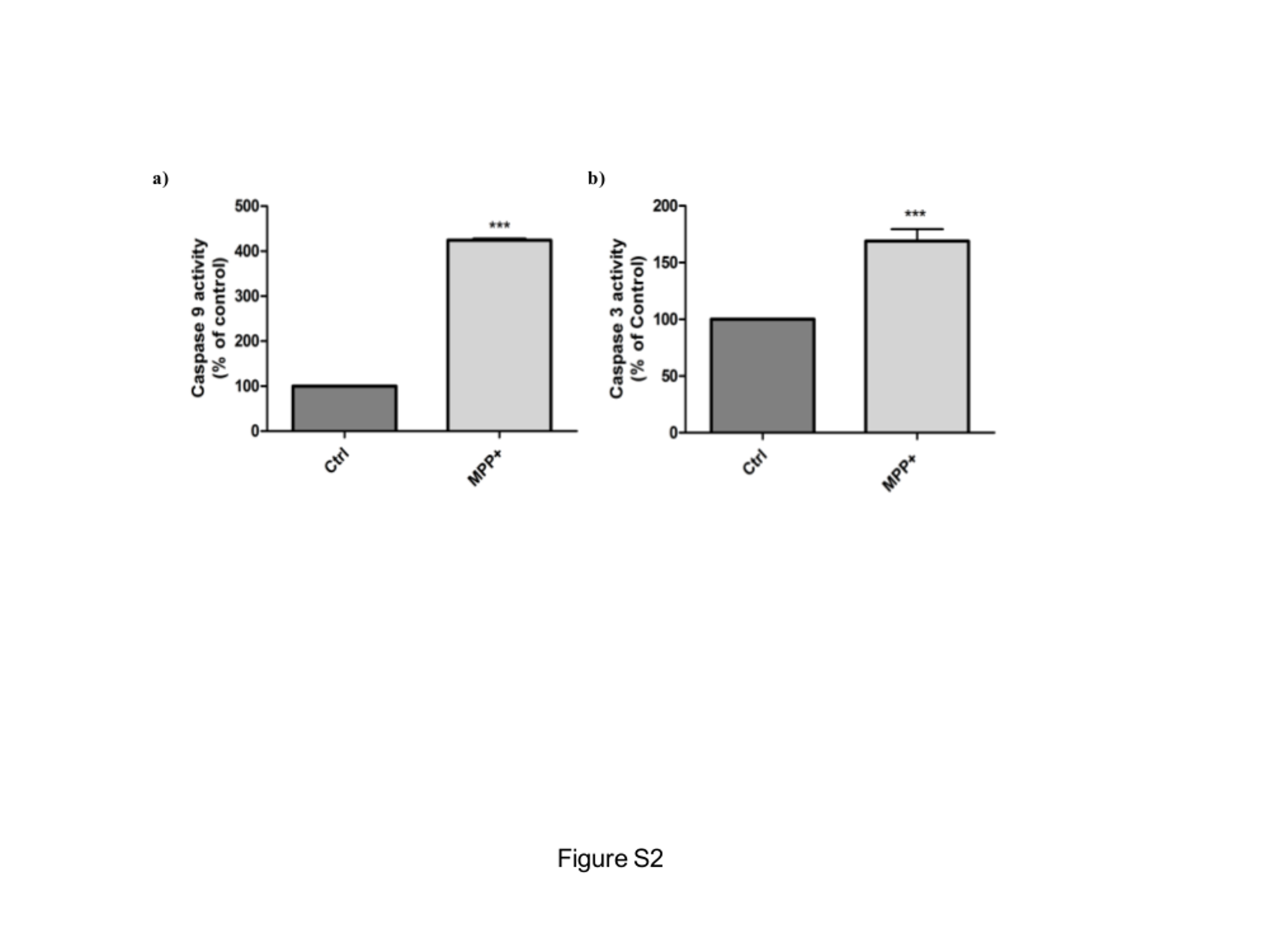

Supplement: S2 Fig — Caspase-9 and -3 activities were measured at 6 and 24 h, respectively after the end of MPP+ treatment, using a specific fluorescent substrate, Ac-LEHD-AFC for caspase-9 (a) and Ac-DEVD-AFC as substrate for caspase-3 (b). Results are expressed as percentage of unstressed control group and represent three independent experiments in triplicate. Results are the mean ± S.E.M. *** p<0.01 compared to control cells. Cells treated under various conditions were harvested through trypsinization and washed with PBS. The cell pellet was gently suspended in buffer containing 30 mM HEPES, 0.3 mM EDTA, 100 mM NaCl, 0.15% Triton X-100 and 10 mM DTT and centrifuged. The supernatant was used for the assay. Caspases substrates were added to a final concentration of 100 mM. The plate was covered, gently mixed and incubated at 37°C for 1 h. Then samples were measured at λex 400 nm and λem 505 nm in a fluorescent microplate reader (TECAN infinite M1000). (TIF) [file pone.0116641.s003.tif]

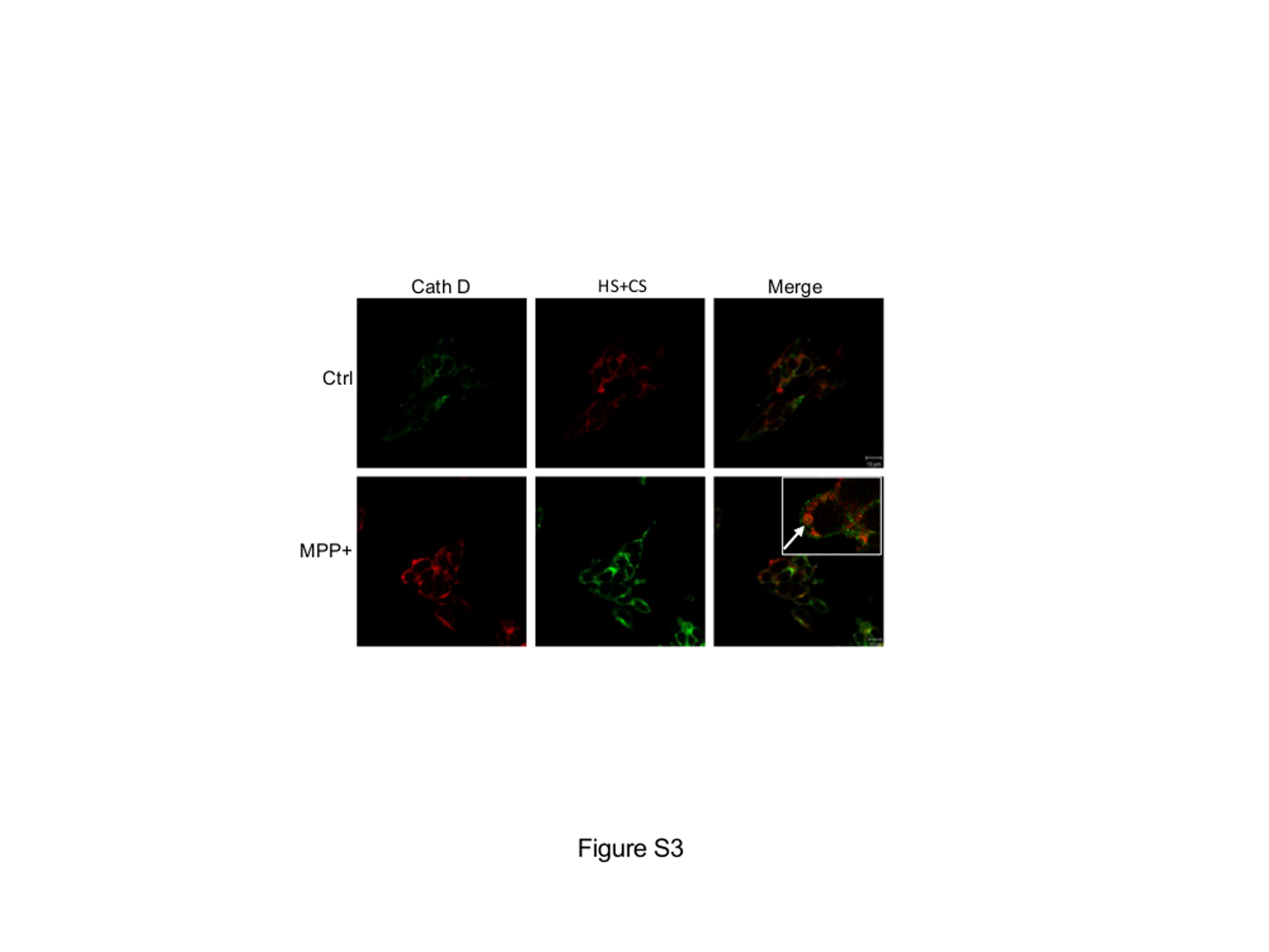

Supplement: S3 Fig — Immunofluorescence co-labeling of cathD (green) and endogenous HS-CS (red) in normal (Ctrl) and MPP+-stressed cells (6 h). Observations were done with a confocal microscope Zeiss Axio Observer Z.1. Stars and arrows indicate areas where co-localization is observed. (TIF) [file pone.0116641.s004.tif]
